# Optical Properties of Nuclear Matter

**DOI:** 10.6028/jres.080A.004

**Published:** 1976-02-01

**Authors:** J. S. O’Connell

**Affiliations:** Institute for Basic Standards, National Bureau of Standards, Washington, D.C. 20234

**Keywords:** Absorption cross section, index of refraction, nuclear matter, optical properties, photon, plasma frequency

## Abstract

The index of refraction and absorptive properties are estimated in nuclear matter consisting of protons and neutrons and in nuclear matter charge neutralized by electrons.

## 1. Introduction

Nuclear matter is a hypothetic substance consisting of interacting neutrons and protons in large enough numbers that the system can be considered infinite. The properties of this medium are determined by the nucleon-nucleon potential (usually with the Coulomb forces between the protons omitted to avoid the infinite Coulomb potential). Two properties, the equilibrium density and binding energy per nucleon, can be derived from measurements on finite nuclei because the conditions at the center of a very large nucleus approximate those of nuclear matter.

The purpose of this investigation is to study the important interaction mechanisms of photons in nuclear matter as a function of frequency in order to estimate the photon propagation characteristics in the medium. In the next section we review some of the basic facts of nuclear matter. In section 3 the photon-nucleon cross sections for various processes are computed. These processes are then reexamined in section 4 for the case where electrons are added to the medium causing net charge neutralization. Finally, a discussion of the results and conclusions are presented in section 5.

## 2. Nuclear Matter

The charge density distribution of finite nuclei as determined by elastic electron scattering indicates the nucleon density of heavy nuclei is fairly constant in the interior of the nucleus with a value

$$\rho =0.170\;\text{nucleons}/{\text{fm}}^{3}.$$

We shall adopt this value for infinite nuclear matter. The total binding energy of finite nuclei as expressed in a semiempirical mass formula can be extrapolated to an infinite number of nucleons (half protons, half neutrons). The average binding energy per nucleon in nuclear matter (i.e., the coefficient of the term linear in number of nucleons) is

B=−15.68MeV/nucleon.

It is the goal of nuclear matter theory to reproduce these “observed” properties, *ρ* and *B*, starting from a potential *V*(**r**) between two nucleons. A review of these calculations is given by Bethe [1]^1^. The Reid soft-core potential, one of the better phenomenological nucleon-nucleon potentials, yields *ρ*_RSC_ = 0.20 fm^−3^ and *B*_RSC_ = −11.25 MeV in a calculation of the two-body contributions. Estimates of higher order contributions seem to account for discrepancies between the Reid potential predictions and the observables.

Nuclear matter is usually discussed within the framework of the Fermi gas model. For no interactions among nucleons (*V*(**r**) = 0) the individual nucleon wave function is a plane wave of momentum **k**

$$\psi \left(r\right)=e^{ik\cdot r}.$$

These functions are normalized in a unit volume and are subject to antisymmetrization for identical nucleons. Our units are chosen to have *ħ* = *c* = 1.

The distribution of nucleon momentum in the Fermi gas model is uniform from *k* = 0 to *k = k_F_* (the Fermi momentum). The maximum momentum *k_F_* is related to the nucleon density by

$$k_{F}^{3}=\frac{3\pi^{2}}{2}\rho$$

which gives *k_F_* =1.36 fm^−1^. This value of the Fermi momentum is also in agreement with inelastic electron scattering in the quasielastic region. This value corresponds to a maximum kinetic energy per particle of

$$\frac{k_{F}^{2}}{2M}=37\;\text{MeV}$$

and an average kinetic energy of 22 MeV. These plane waves form the basis set used in the solution of the problem of interacting nucleons.

In nuclear matter the Schrödinger equation for the wave function *ψ*(*r*) of two nucleons with relative momentum **k** = **k**_1_ − **k**_2_ interacting through a potential *V*(**r**) is modified by a term which takes into account the fact that nucleons cannot scatter into momentum states already occupied by other nucleons. The modification as derived by Bethe and Goldstone for a *s*-wave potential is

$$\left(\frac{d^{2}}{dr^{2}}+k^{2}\right)u\left(r\right)=MV\left(r\right)u\left(r\right)-\frac{1}{\pi }\int_{0}^{\infty }\chi \left(r,r\prime \right)V\left(r\prime \right)u\left(r\prime \right)dr\prime$$

where *u*(*r*) = *kr ψ*(*r*) and

$$\chi \left(r,r\prime \right)=\frac{\sin\;k_{F}\left(r-r\prime \right)}{r-r\prime }-\frac{\sin\;k_{F}\left(r+r\prime \right)}{r+r\prime }.$$

The solution of the Bethe-Goldstone equation can be written as a free wave minus a defect part

u(r)=sinkr−Δu(r).

Figure 2 shows the defect function [2] for the Reid soft-core potential. The function Δ*u*/*k* is not sensitive to either the relative momentum, **k**, or the total momentum **k**_1_ + **k**_2_. An important feature of Δ*u*(*r*) is that it is small by the time *r* approaches the average internucleon separation distance, *ρ*^−^^1/3^ = 1.8 fm. This means that for subsequent collisions nucleons approach each other in relatively pure plane-wave states.

The defect function normalization is such that

$$\kappa =\frac{3}{8}\rho \int_{0}^{\infty }{\left|\Delta u\left(r\right)\right|}^{2}dr$$

is the probability that a *s*-wave pair of particles is outside the Fermi sea. For the Reid soft-core potential *κ* = 0.14 summed over all partial waves.

**Figure 1:**
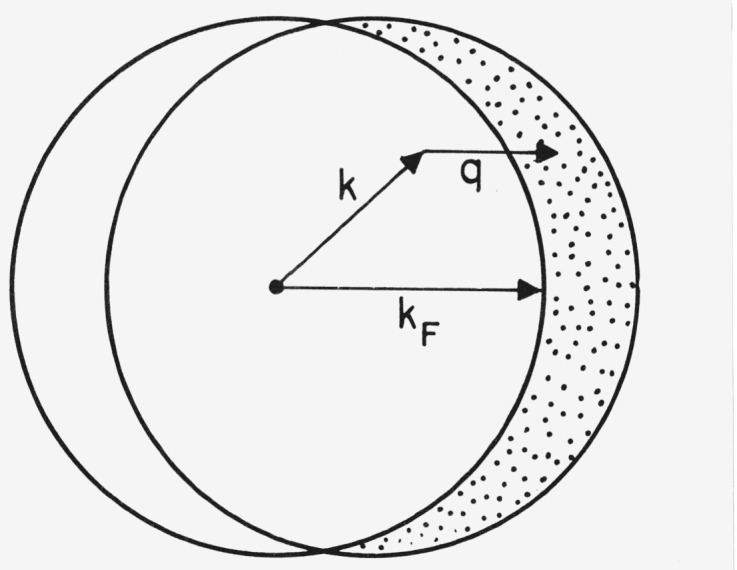
Initial (left sphere) and final (right sphere) momentum distributions in a Fermi gas model in which a momentum *q* is to be given each nucleon. Only those nucleons for which |**k** + **q**| > *k_F_* are allowed to make the transition. The fraction **R**(*q*) is the ratio of excluded volume (shaded) to the total (4/3*π k_F_*^3^).

The Pauli exclusion principle which prevented nucleon-nucleon scattering to occupied momentum states will also modify photon-nucleon interactions. If in free space a photon process imparts a momentum transfer *q* to a nucleon, the cross section in nuclear matter will be reduced by a factor

$$R\left(q\right)=\frac{3}{4}\frac{q}{k_{F}}\left[1-\frac{1}{12}{\left(\frac{q}{k_{F}}\right)}^{2}\right],\;q<2k_{F}$$

because only a fraction *R* of the nucleons will have an initial momentum **k** such that the final momentum will exceed the Fermi momentum |**k** + **q**|>*k_F_*. The expression is derived by calculating the excluded volume of the initial and final Fermi momentum spheres shown in figure 1.

## 3. Photon Interactions

Consider a photon of frequency *ω* incident on a semi-infinite slab of nuclear matter. The index of refraction (ratio of wavelength in vacuum to that in the material) is related to the forward elastic scattering amplitude *f*(*ω*) by

$$n^{2}\left(\omega \right)=1+\frac{4\pi \rho f\left(\omega \right)}{\omega^{2}}.$$

In general, both *f* and *n* are complex numbers. The imaginary part of *f* is related to the absorption cross section through the optical theorem

4πImf(ω)=ωσ(ω).

The real part of *f* can be calculated from the imaginary part by the use of a dispersion relation, but we will not make use of this approach in our estimates. We now examine the photon interaction mechanisms [3].

### 3.1. Thomson Scattering

Electromagnetic waves scatter from the free proton (mass ***M***, charge *e*) with an amplitude

$$f=-\frac{e^{2}}{M}=-1.54\times 10^{-3}\text{fm},$$

which is independent of frequency. The forward amplitude is the same in nuclear matter because no momentum is transferred to the proton. It is convenient to introduce a quantity called the plasma frequency

$$\omega_{0}^{2}=4\pi \rho_{P}\frac{e^{2}}{M},$$

which for *ρ_P_* = 0.085 protons/fm^3^ has the value *ω*_0_ = 8.0 MeV. The index of refraction due to Thomson scattering can be written as

$$n^{2}\left(\omega \right)=1-\frac{\omega_{0}^{2}}{\omega^{2}}.$$

Note that for *ω* < 8 MeV the index *n* is imaginary which means an incident photon will be reflected as it tries to enter the medium. This phenomenon is analogous to the reflection of light waves from the surface of a metal due to Thomson scattering by the conduction electrons.

Within the medium the photon intensity falls off exponentially and the 1/*e* penetration depth is given by

$$d_{\text{Thomson}}={\left(\omega_{0}^{2}-\omega^{2}\right)}^{-1/2}.$$

For *ω* << *ω*_0_ this distance is 25 fm.

Above the plasma frequency *n*(*ω*) is real and the photons propagate freely with the index approaching unity from below.

### 3.2. Proton Compton Effect

Photon scattering by the proton’s charge is inelastic at angles other than 0° and therefore absorptive. The differential cross section for scattering by a free proton is given by the Klein-Nishina formula

$$\frac{d\sigma_{\text{Compton}}^{\text{free}}}{d\Omega }={\left(\frac{e^{2}}{M}\right)}^{2}\frac{1}{2}{\left(\frac{\omega \prime }{\omega }\right)}^{2}\left[\frac{\omega \prime }{\omega }+\frac{\omega }{\omega \prime }-\sin^{2}\theta \right]$$

where the scattered photon energy is

$$\omega \prime =\omega {\left(1+\frac{\omega }{M}\left(1-\cos\;\theta \right)\right)}^{-1}.$$

For incident photon energies small compared to the proton mass, *ω* << *M* = 938 MeV, the photon energy loss *ω* − *ω*′ is small and the free cross section can be approximated by

$$\frac{d\sigma_{\text{Compton}}^{\text{free}}}{d\Omega }≃{\left(\frac{e^{2}}{M}\right)}^{2}\frac{1}{2}\left(1+\cos^{2}\theta \right).$$

In nuclear matter this cross section must be multiplied by the Pauli reduction factor. The momentum transfer to the proton is

$$q=2\omega \;\sin\;\frac{1}{2}\theta .$$

The total Compton cross section for a noninteracting Fermi gas is then

$$\sigma_{\text{Compton}}^{NM\left(\omega \right)}≃\int \frac{3}{4}\frac{q}{k_{F}}\left(\frac{d\sigma^{\text{free}}}{d\Omega }\right)d\Omega =\frac{276}{105}\pi {\left(\frac{e^{2}}{M}\right)}^{2}\frac{\omega }{k_{F}}.$$

For example, $\sigma_{\text{Compton}}^{NM}\left(100\;\text{MeV}\right)=0.73\times 10^{-31}{\text{cm}}^{2}$.

### 3.3. Pair Production

An important absorption mechanism is the conversion of the photon to an electron-positron pair in the Coulomb field of the proton. The free proton cross section for *ω* >> *m_e_* is given by

$$\sigma_{\text{pair}}^{\text{free}}={\left(\frac{e^{2}}{m_{e}}\right)}^{2}\left[\frac{28}{9}\ln\frac{2\omega }{m_{e}}-\frac{218}{27}\right]$$

where *m_e_* is the mass of the electron. The proton is required to take up an average momentum transfer of $\bar{q}≅m_{e}$. Thus, the nuclear matter cross section per proton is

$$\sigma_{\text{pair}}^{{\text{NM}}_{\left(\omega \right)}}=\frac{3}{4}\frac{m_{e}}{k_{F}}\sigma_{\text{pair}}^{{\text{free}}_{\left(\omega \right)}}.$$

A 1/*e* absorption length can be computed using

$$d={\left(\rho_{P}\sigma \right)}^{-1}.$$

Typical values are:

**Table:** 

*ω* (MeV)	$\sigma_{\text{pair}}^{\text{NM}}\left(\times 10^{-27}{\text{cm}}^{2}\right)$	Imf_pair_ (×10^−3^fm)	*d*_pair_ (fm)
10.	0.38	0.15	306.
30.	0.77	0.93	153.
100.	1.19	4.80	99.

The imaginary part of the forward scattering amplitude exceeds the real Thomson aplitude above 50 MeV.

### 3.4. Absorption on Correlated Neutron-Proton Pairs

Single particle ejection, which is an important photoabsorption mechanism in finite nuclei, is not possible in a noninteracting system in which the nucleon has definite linear momentum because energy and momentum cannot be simultaneously conserved. However, in an interacting Fermi gas the defect wave function has a spread of momentum components that allows two nucleons to absorb a photon and be ejected from the Fermi sea.

An estimate of the absorption cross section of this process can be made by considering the electric dipole transition of a correlated neutron-proton pair using the *s*-wave defect wave function as the initial state and a free *p*-wave for the final state

$$do={\left(2\pi \right)}^{2}\frac{e^{2}}{M^{2}\omega }{\left|ℳ\left(p\right)\right|}^{2}\frac{dn}{d\omega }.$$

The density of final two-nucleon states is a function of the final relative momentum **p**

$$\frac{dn}{d\omega }=\frac{d^{3}p}{{\left(2\pi \right)}^{3}d\omega }=\frac{1}{{\left(2\pi \right)}^{3}}\frac{Mp}{2}d\Omega_{p}.$$

The *E*1 matrix element is calculated from the momentum space wave functions as

$$ℳ\left(p\right)=\int \psi_{f}\hat{\epsilon }\cdot k\;\psi_{i}d^{3}k$$

where $\hat{\epsilon }$ is the photon polarization vector.

A properly normalized initial state is

$$\psi_{i}\left(k\right)={\left(3\rho /8\right)}^{1/2}\cdot \Delta \psi \left(k\right)$$

where for convenience we use the Fourier transform of the defect wave function

$$\Delta \psi \left(k\right)=\frac{1}{{\left(2\pi \right)}^{3/2}}\int e^{ik\cdot r}\frac{\Delta u\left(r\right)}{kr}d^{3}k.$$

The final state with both particles outside the Fermi momentum sphere is

$$\psi_{f}={\left(2\pi \right)}^{3/2}\delta^{3}\left(\text{p-k}\right).$$

The matrix element is simply evaluated as

$$ℳ\left(p\right)={\left(3\;\rho /8\right)}^{1/2}{\left(2\pi \right)}^{3/2}\hat{\epsilon }\cdot p\Delta \psi \left(p\right).$$

The total cross section averaged over photon polarization is then

$$\sigma \left(\omega \right)=\pi^{3}\frac{e^{2}}{M}\rho \left(\frac{p^{3}}{\omega }\right){\left|\Delta \psi \left(p\right)\right|}^{2}.$$

We take the threshold for the reaction to be twice the average single particle binding energy, 16 MeV. Thus, photon energy and final relative momentum are related by

$$\omega =\frac{p^{2}}{M}+32\;\text{MeV}.$$

**Figure 2:**
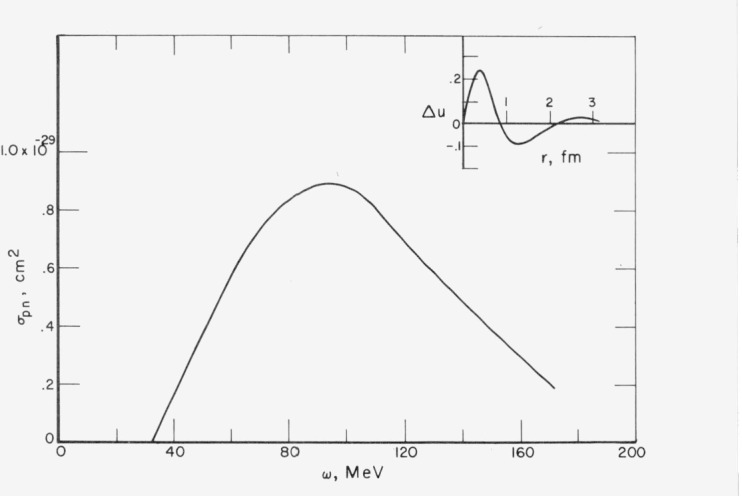
Upper right insert shows *s*-wave defect wave function for a soft-core nucleon-nucleon potential. The curve *σ_pn_* is the cross section per nucleon for the absorption of a photon by a correlated neutron-proton pair calculated using Δ*u*.

The resulting cross section is plotted in figure 2. At the peak *ω* = 100 MeV the nuclear matter cross section is about 13 percent of the free deuteron cross section.

### 3.5. Pion Production

At photon energies greater than the mass of the pion *ω* > *m_π_* = 140 MeV the reaction

γ+N→N+π

occurs for both neutrons and protons. The cross section for free nucleons reaches a peak at *ω* = 300 MeV of 0.5 × 10^−27^ cm^2^ (the 3, 3 resonance). After a few smaller resonances the cross section is essentially constant between 2 GeV and 20 GeV at 0.1 × 10^−27^ cm^2^. One might expect that the nuclear matter cross section would be the free cross section modified by the Pauli reduction factor *R*(*q*) where the nucleon recoil momentum is

$$q={\left[\frac{2m_{\pi }M}{M+m_{\pi }}\left(\omega -m_{\pi }\right)\right]}^{1/2}.$$

However, the *A* dependence of the high energy absorption cross section for finite nuclei

$$\frac{\sigma_{\gamma A}}{\sigma_{\gamma N}}=\{\begin{array}{cc} A^{0.75}, & \;\omega =0.14\;\text{to}\;2\;\text{GeV}\\ A^{0.91}, & \omega >2\;\text{GeV} \end{array}$$

indicates [4] a shadowing phenomenon which is more complicated than the incoherent sum of individual nucleon events in nuclear matter. Present theories contend that a high energy photon spends a fraction of the time as a spin-one meson (vector dominance model). Mesons propagating in nuclear matter have a large and complex index of refraction [5]. For our purposes, however, it is probably safe to assume that the photo pion absorption cross section per nucleon is no larger than that of the free nucleon at the same photon energy. In this case, the high energy absorption will be dominated by the ever-increasing *e*^+^*e*^−^ production process.

## 4. Photon Interactions in Charge Neutralized Nuclear Matter

If electrons are added to the proton-neutron matter to achieve charge neutrality (*ρ_e_* = *ρ_P_* = 0.085 fm^−3^) the optical properties are drastically changed. Müller and Rafelski [6] argue that charge neutralization occurs spontaneously when the Coulomb potential exceeds ${\left[k_{F^{2}}+m_{e}\right]}^{1/2}≃k_{F}=268\;\text{MeV}$. This occurs for finite nuclei which have more than 10^4^ protons.

The plasma frequency of the electrons is much higher than that of the protons because of the smaller electron mass,

$$\omega_{0}^{\text{electron}}={\left(4\pi \;\rho_{e}\;e^{2}/m_{e}\right)}^{1/2}=342\;\text{MeV},$$

with an associated penetration depth less than a fm. As a consequence, any photons with energy less than 342 MeV will be reflected from the surface of a semiinfinite slab of this material.

Photon propagation in the medium becomes possible again for *ω* > 342 MeV. Pair production in the Coulomb fields of the electrons and protons is influenced by the requirement that the *e*^−^ which is produced must have a momentum greater than *k_F_*. Thus, the threshold for pair production is raised from 2*m_e_* to *k_F_* and the $\ln\frac{2\omega }{m_{e}}$ term in the high energy cross section formula becomes $\ln\frac{2\omega }{k_{F}}$. In the threshold region the *e*^+^ and *e*^−^ energies are not shared symmetrically and there will be mutual screening of the electron and proton Coulomb fields. An estimate of the combined pair production cross section can only be made for *ω* > 2 GeV, viz.,

$$\sigma_{\text{pair}}^{\text{epn}}\left(\omega \right)≃\frac{3}{2}\frac{m_{e}}{k_{F}}{\left(\frac{e^{2}}{m_{e}}\right)}^{2}\left[\frac{28}{9}\ln\frac{2\omega }{k_{F}}-\frac{218}{27}\right].$$

The photo pion absorption cross section is unaffected by the electron component of nuclear matter. Above 2 GeV the pair cross section is larger than $\sigma_{\gamma_{\pi }}$, but the reverse is probably true between 342 MeV and 2 GeV. Compton scattering on the electrons is smaller than both these cross sections.

## 5. Discussion and Conclusions

**Figure 3:**
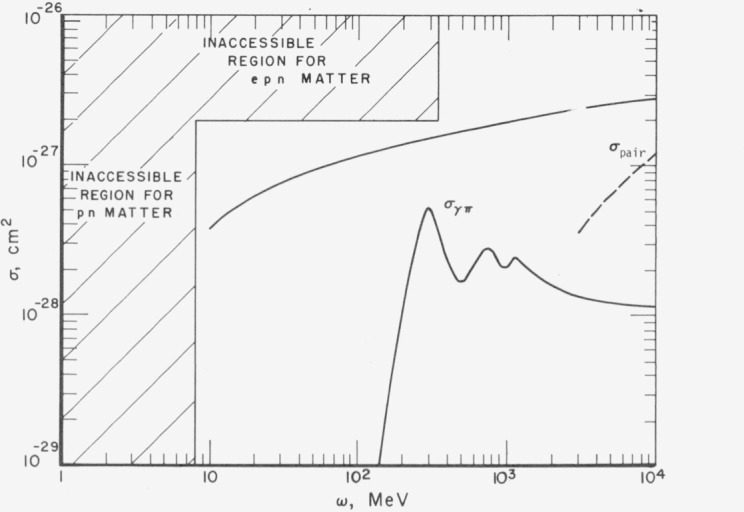
Cross sections for dominant photoabsorption processes in nuclear matter. The vertical line at *ω* = 8 MeV marks the frequency below which photons will not propagate in matter composed of protons and neutrons. The line at *ω* = 342 MeV is the limiting energy for matter composed of electrons, protons, and neutrons. The solid curve labeled *σ*_pair_ is the absorption cross section per proton for *e*^+^*e*^−^ production in *pn* matter: the dashed curve for *epn* matter. The curve labeled $\sigma_{\gamma_{\pi }}$ is the free nucleon photo pion absorption cross section. This cross section is probably an upper limit to the process in nuclear matter Other photon interaction cross sections, Compton scattering, and nucleon pair absorption, are less that 10^−29^cm^2^ at all energies.

The main results are displayed in figure 3. Protonneutron matter is dominated by reflective Thomson scattering below 8 MeV and pair production above the proton plasma frequency. All real nuclei are too small to exhibit the low frequency imaginary index of refraction expected of *np* matter since the largest diameter (14 fm) is still small compared to the penetration depth (25 fm). Also, pair production on finite nuclei occurs coherently in the intense Coulomb field at the edge of the nucleus rather than from individual protons in the interior.

Electron-proton-neutron matter has a much higher plasma frequency so that photons do not penetrate the medium until *ω* > 342 MeV. Pion production from the protons and neutrons dominates the absorption up to about 2 GeV, then pair production becomes more important; however, both these processes are difficult to calculate accurately.

## Footnotes

1 Figures in brackets indicate the literature references at the end of this paper.
